# Association Between Telemedicine Use in Nonmetropolitan Counties and Quality of Care Received by Medicare Beneficiaries With Serious Mental Illness

**DOI:** 10.1001/jamanetworkopen.2022.18730

**Published:** 2022-06-27

**Authors:** Bill Wang, Haiden A. Huskamp, Sherri Rose, Alisa B. Busch, Lori Uscher-Pines, Pushpa Raja, Ateev Mehrotra

**Affiliations:** 1 Department of Health Care Policy, Harvard Medical School, Boston, Massachusetts; 2 Stanford University, Stanford, California; 3 McLean Hospital, Belmont, Massachusetts; 4 RAND Health, Arlington, Virginia; 5 Veterans Affairs Greater Los Angeles Healthcare System, Los Angeles, California

## Abstract

**Importance:**

Access to specialty mental health care remains challenging for people with serious mental illnesses, such as schizophrenia and bipolar disorder. Whether expansion of telemedicine is associated with improved access and quality of care for these patients is unclear.

**Objective:**

To assess whether greater telemedicine use in a nonmetropolitan county is associated with quality measures, including use of specialty mental health care and medication adherence.

**Design, Setting, and Participants:**

In this cohort study, the variable uptake of telemental health visits was examined across a national sample of fee-for-service claims from Medicare beneficiaries in 2916 nonmetropolitan counties between January 1, 2010, and December 31, 2018. Beneficiaries with schizophrenia and related psychotic disorders and/or bipolar I disorder during the study period were included. For each year of the study, each county was categorized based on per capita telemental health service use (none, low, moderate, and high). The association between telemental health service use in the county and quality measures was tested using a multivariate model controlling for both patient characteristics and county fixed effects. Analyses were conducted from January 1 to April 11, 2022. Before the COVID-19 pandemic, telemedicine reimbursement was limited to nonmetropolitan beneficiaries.

**Main Outcomes and Measures:**

Receipt of a minimum of 2 specialty mental health service visits (telemedicine or in-person) in the year, number of months per year with medication, hospitalization rate, and outpatient follow-up visits after a mental health hospitalization in a year.

**Results:**

In 2018, there were 2916 counties with 118 170 patients (77 068 [65.2%] men; mean [SD] age, 58.3 [15.6] years) in the sample. The fraction of counties that had high telemental health service use increased from 2% in 2010 to 17% in 2018. In 2018 there were 1.08 telemental health service visits per patient in the high telemental health counties. Compared with no telemental health care in the county, patients in high-use counties were 1.2 percentage points (95% CI, 0.81-1.60 percentage points) (8.0% relative increase) more likely to have a minimum number of specialty mental health service visits, 13.7 percentage points (95% CI, 5.1-22.3 percentage points) (6.5% relative increase) more likely to have outpatient follow-up within 7 days of a mental health hospitalization, and 0.47 percentage points (95% CI, 0.25-0.69 percentage points) (7.6% relative increase) more likely to be hospitalized in a year. Telemental health service use was not associated with changes in medication adherence.

**Conclusions and Relevance:**

The findings of this study suggest that greater use of telemental health visits in a county was associated with modest increases in contact with outpatient specialty mental health care professionals and greater likelihood of follow-up after hospitalization. No substantive changes in medication adherence were noted and an increase in mental health hospitalizations occurred.

## Key Points

**Question:**

Is there an association between greater telemedicine use in a nonmetropolitan county and quality measures including use of specialty mental health care and medication adherence?

**Findings:**

In this cohort study including 118 670 patients with schizophrenia or related psychotic disorders and/or bipolar I disorder, greater use of telemental health visits in a nonmetropolitan county was associated with modest increases in contact with outpatient specialty mental health professionals and greater likelihood of follow-up after hospitalization. However, there were no substantive changes in medication adherence, and there was an increase in mental health hospitalizations.

**Meaning:**

The findings of this study suggest that greater use of telemental health service may be associated with quality measures.

## Teaser

This cohort study examines the use of telemental health visits by persons with serious mental illness in nonmetropolitan counties across the US.

## Introduction

Patients with severe mental illness (SMI) often face difficulty accessing mental health specialty care. This hindrance is particularly true in nonmetropolitan areas where the shortage of health care professionals is acute and the rates of specialty mental health care visits per person are roughly half those seen in urban areas.^1,2,3^ Telemental health interaction is one potential solution for these access barriers. Before the COVID-19 pandemic, there was rapid growth in the number of live video-based telemental health service visits in nonmetropolitan areas.^4,5^ Since the start of the COVID-19 pandemic, that growth has accelerated, and in some analyses most visits with behavioral health care professionals are via telemedicine.^6^ Although numerous randomized clinical trials have demonstrated that live-video telemental health and in-person visits are comparable in treating patients with mental illness,^7,8,9^ it has never been demonstrated that greater use of telemental health visits outside of clinical trials improves care quality by expanding access to needed care. Our goal in this study was to test whether greater use of telemental health service would be associated with increased engagement (measured by total number of interactions) with specialty mental health care professionals at the population level and lead to improvements in other components of mental health care delivery and quality (specifically, reductions in emergency department [ED] visits and hospitalizations, improvement in postdischarge outpatient mental health engagement, and improved medication adherence).

These analyses are timely because there is active debate in many states on whether to extend pandemic-related temporary expansions of telemedicine for mental illness care. Allowing greater flexibility to use telemedicine on a permanent basis (eg, without originating or geographic restrictions) is advisable only if it can improve access and/or improve care quality.^10^ To help inform this debate, we examined data from the prepandemic period and compared changes over time in care in nonmetropolitan counties with high vs low telemental health service use. We focused on people with schizophrenia and other psychotic disorders or bipolar I disorder given that these are among the most severe mental health conditions. Furthermore, this population with SMI is most in need of specialty mental health care and has the highest use of telemental health visits.^11^

## Methods

### Overview

In this cohort study, we used Medicare fee-for-service data and focused only on beneficiaries in nonmetropolitan communities, because before the pandemic Medicare coverage of telemedicine was largely limited to nonmetropolitan communities. Our unit of analysis is the patient year.

To estimate the value of telemental health visits, we examined the variable uptake of telemental health visits across nonmetropolitan counties. Each year, each county was categorized based on per capita telemental health service use among patients with SMI (no use, low use, moderate use, high use, as defined in the Identifying and Classifying Mental Health Visits section). We tested the association between use of telemental health visits at the county level instead of whether the individual patient had a telemental health service visit. This analytic strategy was used to address the potential for patient selection bias, that is, patients who use telemedicine may be systematically different in a manner that cannot be captured with measurable characteristics and these systematic differences could affect differences in our outcomes. Our models also included a separate variable for each county in the US. In such a fixed-effect analysis, we estimated the changes in outcomes within a county as the use of telemental health changes (eg, county goes from low use to high use). The exposure for a patient is the county’s use of telemental health in that year regardless of whether that patient received those services. This study followed the Strengthening the Reporting of Observational Studies in Epidemiology (STROBE) reporting guideline for observational studies. The Harvard Medical School Institutional Review Board exempted this study from review and the requirement for informed consent because the data were previously collected and deidentified.

### Data Source

We used Medicare claims from January 1, 2010, to December 31, 2018, for a 20% sample of fee-for-service patients with continuous Part A and B Medicare coverage in a given year who lived in a nonmetropolitan area. These data encompassed adults older than 65 years as well as 8.8 million adults who entered the program at a younger age owing to disability. Consistent with the Medicare definition of nonmetropolitan eligibility for telemedicine reimbursement,^12^ we defined nonmetropolitan patients as those residing in zip codes outside of a core-based statistical area or within an area assigned a nonmetropolitan urban commuting area code of 4 (micropolitan areas) to 10 (nonmetropolitan areas). To address the fact that some counties have very few residents, we excluded counties that did not have at least 1 visit for patients with SMI in every study year. Our final sample encompassed 2916 nonmetropolitan counties.

### Patient Cohorts

We developed a retrospective cohort of individuals with SMI. To be in our cohort, patients must have had 2 outpatient visits with a diagnosis of schizophrenia or a related psychotic disorder (*International Classification of Diseases, Ninth Revision* [*ICD-9*] codes 295, 297; *International Statistical Classification of Diseases, Tenth Revision* [*ICD-10*] codes F20.x-F29.x) and/or bipolar I disorder (*ICD-9* codes 296.0, 296.1, 296.4-296.6, 296.7; *ICD-10* codes F30.x, F31.0-F31.7, F31.9) in any diagnosis field or 1 inpatient claim with these diagnoses in the first or second diagnosis field during any point between 2010 and 2018. Instead of recreating the cohort each year based on care patterns, we chose to include patients in the cohort regardless of whether they had a visit in a given year. These disorders typically have onset early in adulthood and are lifelong illnesses. We did not want to exclude patients for whom access or personal barriers may have resulted in not having any visits in a given calendar year. Patients who moved during the sampling period were assigned to the county of residence during a given patient year. Patients were only included for years that they were enrolled in Medicare fee for service. As a sensitivity analysis, we also developed a prospective cohort in which a patient was included in the cohort only in years after a diagnosis of SMI. In addition to diagnosis, we obtained data on age, sex, and race and ethnicity for demographic information.

We used a hierarchical method when dividing the cohort into individuals with schizophrenia or a related psychotic disorder vs bipolar I disorder. Those with schizophrenia or a related psychotic disorder were individuals with at least 2 outpatient visits or 1 inpatient hospitalization for that diagnosis between 2010 and 2018. The remainder of the cohort was categorized as having bipolar I disorder.

### Identifying and Classifying Mental Health Visits 

Consistent with earlier work, we identified mental health–related visits as those with a mental health diagnosis in the first or and second diagnosis field (eTable 3 in the Supplement).^11,13,14^ We focused on visits to specialty mental health care professionals, which we defined as visits to a psychiatrist, psychologist, clinical psychologist, clinical social worker, or nurse practitioner who specializes in mental illness (specialty codes 26, 86, 62, 68, and 80). Also consistent with earlier work, nurse practitioners were identified as mental health specialists based on the types of conditions they treat.^15^ A telemedicine visit was defined as a visit with a GT (via interactive audio and video telecommunication systems) or GQ (via asynchronous telecommunications system) Medicare modifier code.^14^ During this period, audio-only telemedicine visits were not reimbursed.

Across all county-years, we determined the median and 90th percentile of telemental health service visits per 100 patients with SMI. We categorized counties each year into 4 groups: no telemental health service use, low (>0 to median), moderate (visit rate median ≥10.6 visits per 100 patients with SMI but less than 90th percentile), and high (telemental health service use ≥90th percentile of 29.7 telemental health service visits per 100 patients with SMI in a year). The no use group had 0 visits per SMI patient and served as the controls.

### Other County Characteristics

We compared county characteristics between uptake groups, using variables from the 2010 US Census and 2019 Area Health Resource File. As proxies for health care access, we examined median household income, population density, broadband access, and access to hospital beds, community health centers, physicians, and nurses (eTable 2 in the Supplement).

### Quality Measures

First, we examined whether patients in the county received a minimum number of specialty mental health service visits (either via telemedicine or in person). Our hypothesis was that telemedicine would lead to more patients receiving minimum levels of contact with specialty mental health care professionals. Second, we focused on medication adherence, outpatient follow-up visits after a mental health hospitalization, and acute care use (ED and hospitalization). Our hypothesis was that more contact with specialty mental health care professionals would be associated with greater medication adherence, less acute care use, and if there is a hospitalization, greater likelihood of follow-up care after the hospitalization.

In each patient year, we focused on visits (either in person or telemedicine) to mental health care specialty clinicians. We focused on specialty care because schizophrenia and bipolar I disorder typically need specialty management. We measured what fraction of the cohort had at least 1 mental health specialty visit in the first half and the second half of the year as a minimum threshold of engagement; the Veterans Affairs national health system has added a similarly structured performance measure to its national evaluation systems used for mental health care quality management.^15,16^ Because of concerns that 2 visits could be too low a threshold for sufficient use for patients with schizophrenia and bipolar I disorder, we also measured what fraction of patients visited a mental health specialist at least once per calendar quarter.^17^

We measured the number of months with days of supply of medication available, a National Quality Forum–sponsored measure.^18^ For patients with schizophrenia or related psychotic disorders, we focused on antipsychotic medications. For patients with bipolar I disorder, we measured adherence to either an antipsychotic or mood-stabilizing medication (eTable 1 in the Supplement). Consistent with a National Committee for Quality Assurance measure, we captured what fraction of patients with mental health hospitalizations had an outpatient mental health service visit within 7 or 30 days of discharge.^19^

In addition, we measured mental health–specific acute care use (ie, ED visit and hospitalization) in a given patient year. Emergency department visits and hospitalizations were classified as mental health specific if the primary diagnosis was a mental health diagnosis. We recognize that acute care use is not typically used as a measure of quality, since many exogenous factors (eg, local bed supply, off-hours mental health staffing) could affect acute care use and that, for a given patient, hospitalization or an ED visit could be necessary. However, consistent with the concept of the Agency for Healthcare Research and Quality potentially avoidable hospitalization measures,^20^ we believed that, at a population level, there might be a reduction in mental health–related ED visits and hospitalizations in a county among patients with SMI if patients receive more beneficial outpatient mental health care.

### Subgroup Analyses

We performed subgroup analysis including only patients dually eligible for Medicaid and another excluding these patients. Medicare does not reimburse visits from licensed professional counselors or licensed marriage and family therapists^21^ and in some states individuals with dual eligibility may have those visits covered by Medicaid and would not be captured in our data. We also stratified the population by age (<65 vs ≥65 years) to assess whether there were age-related differences in the association between telemedicine use and our outcomes.

### Statistical Analysis

Each county in each year was divided into 4 groups based on telemedicine use during that calendar year. We used linear regression that accounted for the clustering of patients within the county. We created dummy variables for the level of telemedicine uptake in the county that year (high_i_, moderate_i_, low_i_), excluding the reference group of no telemental health uptake. We regressed each quality measure (*Y_i_*) against the intervention (level of telemedicine uptake), including dummy variables for each year and county (also termed fixed effects) (eFigure in the Supplement). The county-level fixed effects capture the observed and unobserved time-invariant county level covariates, whereas the variables for each year capture time trends. Given the structure of the model, we did not add covariates for individual characteristics (eg, comorbid conditions) under the assumption that the prevalence of chronic conditions is similar across years in a given county (eFigure in the Supplement). The coefficients *high_i_, moderate_i_,* and *low_i_* that we observed represent the relative effect on a quality measure of a county switching from the no telemedicine group to that level of telemedicine uptake. Analyses were conducted from January 1 to April 11, 2022. All statistical analyses were performed using SAS, version 9.4 (SAS Institute Inc). Statistical analysis was done with 2-sided hypothesis tests, with α = .05 as the significance threshold.

## Results

There were 2916 counties in our sample with a total of 118 170 patients (77 068 [65.2%] men; mean [SD] age, 58.3 [15.6] years). In 2018, the last year of the study period, these counties were categorized as follows: no telemental health service use (1459 counties, 37 428 patients with SMI), low telemental health service use (516 counties, 40 570 patients with SMI), moderate telemental health service use (438 counties, 23 504 patients with SMI), and high telemental health service use (503 counties, 17 168 patients with SMI). Patient characteristics were generally similar across these 4 groups of counties (Table 1). For example, 78.4% of patients in the high telemental health service visits group and 77.9% in the no telemental health service visits group were younger than 65 years.

**Table 1.** Characteristics of Patients With Severe Mental Illness Stratified by Use of Telemental Health Service in Nonmetropolitan Counties, 2018

Variable	County categories^a^				*P* value for difference across counties^b^
	None (0)	Low (0-0.11)	Moderate (0.11-0.30)	High (>0.30)	
No. of counties	1459	516	438	503	NA
No. of patients with SMI	37 428	40 570	23 504	17 168	NA
Patient characteristics					
Race and ethnicity, %					
Non-Hispanic					
White	83.5	83.7	84.5	82.8	<.001
Black	10.2	10.3	8.3	8.9	<.001
Hispanic	3.2	3.1	3.7	5.2	<.001
Other^c^	3.0	2.9	3.4	3.2	<.001
Age, y					
<65	77.9	78.7	78.7	78.4	.02
65-74	12.6	12.2	12.4	12.5	.28
75-84	6.5	6.4	6.2	6.1	.28
>84	3.0	2.8	2.8	3.0	.11
Sex					
Female	33.8	34.8	35.2	36.5	<.001
Male	66.2	65.2	64.8	64.5	
Type of mental illness					
Schizophrenia/psychotic disorders	38.4	37.4	38.7	40.1	<.001
Bipolar I	61.6	62.6	61.3	59.9	
Dual eligible for Medicaid	69.0	70.5	70.9	73.1	<.001
Qualified for Medicare based on age	21.7	19.8	19.2	18.9	<.001
County characteristics					
Household income, median, $	43 583	42 208	41 446	41 004	<.001
Population density, person/square mile	114.67	94.77	77.12	70.7	<.001
Household with broadband, %	79.0	78.9	79.8	80.3	<.001
Hospital Beds per 1000 people in county	3.7	2.1	2.5	2.2	<.001
Community health centers per 1000 people in county	2.4	1.7	1.7	1.8	<.001
Physicians/advanced practice nurses per 100 people in county	2.8	1.2	1.7	1.1	<.001

Abbreviations: NA, not applicable; SMI, serious mental illnesses.

^a^ Telemental health service visits per patient with serious mental illness in the county in 2018.

^b^ Differences tested with analysis of variance.

^c^ We combined 4 Medicare-designated categories: American Indian or Alaska Native, Asian or Pacific Islander, other, and unknown.

In 2018, counties with high telemental health service visits were less densely populated (70.7 persons/sq mile) than those with no telemental health service visits (114.7 persons/sq mile). In 2018, high telemental health service visits counties also had fewer health care professionals (mean, 2.2 hospital beds per 1000 people in the county, 1.1 physician or advanced practice nurse per 1000 people in the county) than the no telemental health service visits counties (mean, 3.7 hospital beds, 2.8 physicians or advanced practice nurses).

### Changes in Telemental Health Service Use

**Figure 1.**
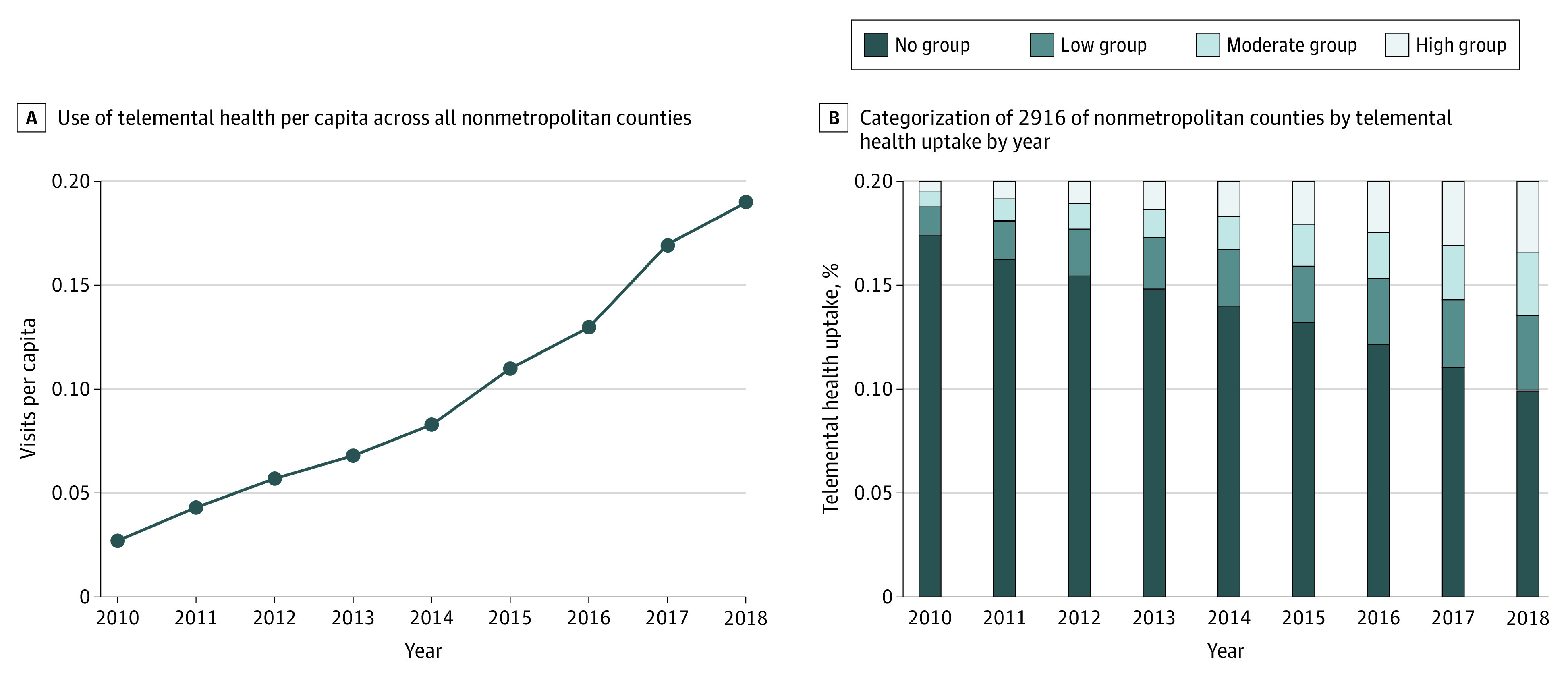
Telemental Health Service Outcomes A, Use of telemental health service per capita across all nonmetropolitan counties in the sample. B, Categorization of 2916 nonmetropolitan counties by telemental health service uptake by year.

In 2010, across all patients with SMI in all the nonmetropolitan counties in the sample, telemental health service use was 0.03 visits per patient with SMI; in 2018, telemental health service use was 0.19 visits per patient with SMI (Figure 1A). Accordingly, as of 2018, more counties were categorized in the moderate and high telemental health service use groups; for example, in 2010, 2% of counties were in the high-use group and, in 2018, 17% were in the high-use group (Figure 1B).

### Changes in Total Specialty Mental Health Visits

**Figure 2.**
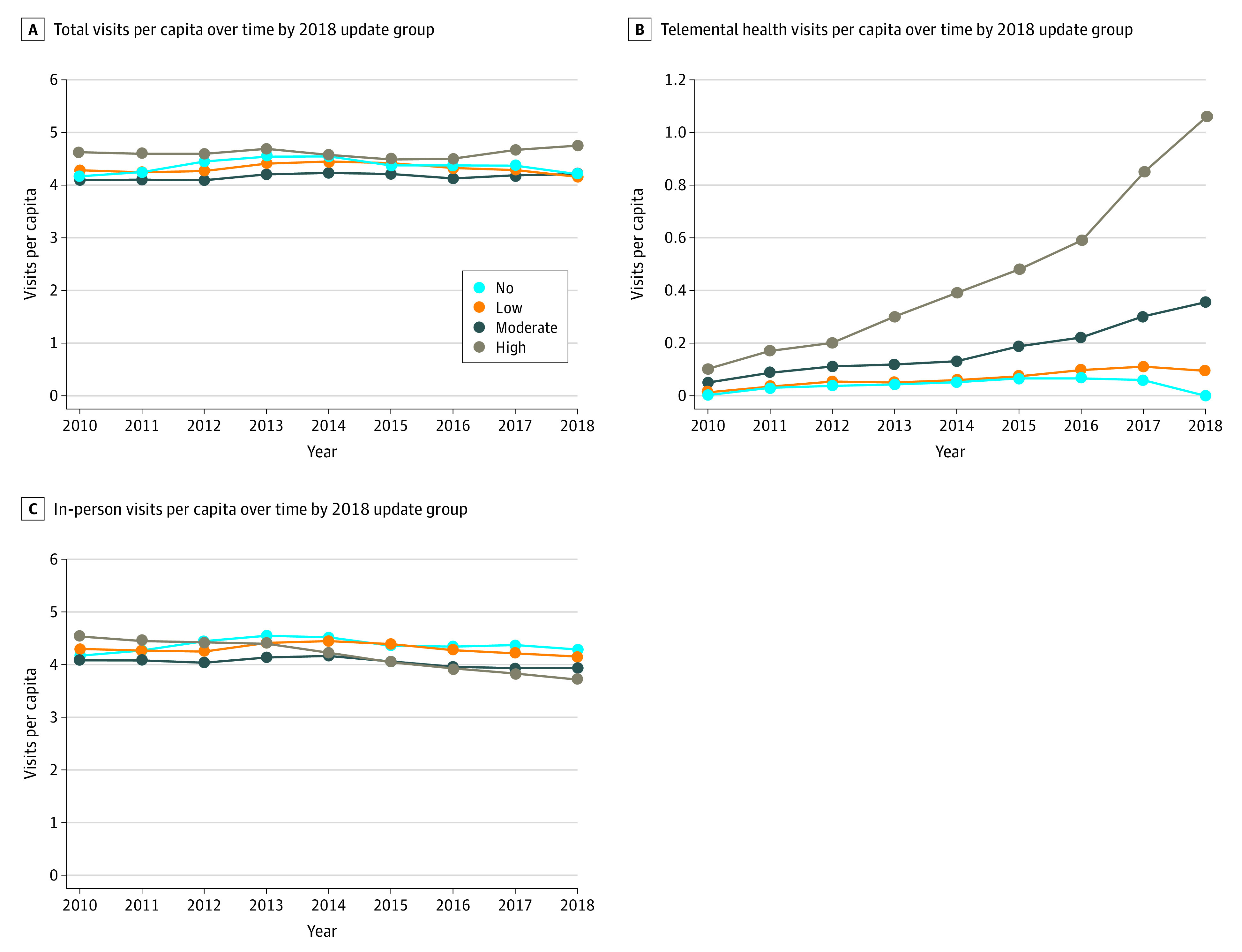
Mental Health Visits Per Capita of Nonmetropolitan Counties Grouped by Telemental Health Service Uptake in 2018 The value for none is 0; the value range for low is 0 to 0.11; moderate, 0.11 to 0.30; and high, greater than 0.30.

In 2010, counties with no telemental health service use had 4.22 total (in-person or telemedicine) mental health service visits per patient. In those same counties, the number of visits per patient in 2018 was slightly higher (4.29) (Figure 2). In counties with high telemental health service use in 2018, in-person visits decreased from 4.55 (2010) to 3.73 (2018). However, owing to the increase in telemental health service use in these counties (1.06 visits per capita), overall visits (in-person plus telemental health service) increased modestly, from 4.65 (2010) to 4.79 (2018) (Figure 2).

### Changes in Likelihood of Receiving Other Forms of Utilization

In our main model, compared with patients in the no telemental health service use counties, patients in high telemental health service use counties were 1.2 percentage points (95% CI, 0.81-1.60 percentage points) (8.0% relative increase) more likely to have a minimum of 4 specialty mental health service visits, 2.2 percentage points (95% CI, 5.1-22.3 percentage points) (6.5% relative increase) more likely to have outpatient follow-up within 7 days of a mental health hospitalization, and 0.47 percentage points (95% CI, 0.25-0.69 percentage points) (7.6% relative increase) more likely to be hospitalized in a year (Table 2). There was no significant association between telemental health service use and months of medication treatment.

**Table 2.** Association Between Telemental Health Use in Nonmetropolitan Counties and Outcomes for Patients With Serious Mental Illness

Variable	County categories^a^						
	None (0)	Low (0-0.11)		Moderate (0.11-0.30)		High (>0.30)	
	Reference rate in 2010 in no use, %	% Difference compared with reference group (95% CI)	% Change relative to no use group	% Difference compared with reference group (95% CI)	% Change relative to no use group	% Difference compared with reference group (95% CI)	% Change relative to no use group
Visit patterns, ≥1 visits %							
Per quarter in year	15.1	0.4 (0.1 to 0.6)	2.3	0.02 (−0.30 to 0.33)	0.1	1.2 (0.8 to 1.6)	8.0
Per semester in year	27.7	0.4 (0.2 to 0.6)	1.4	0.7 (0.4 to 1.1)	2.5	1.3 (0.9 to 1.8)	4.7
Follow-up after hospitalization, %							
7 d	34.0	−0.3 (−2.0 to 1.5)	−0.8	1.1 (−1.5 to 3.7)	3.2	2.2 (−1.0 to 5.5)	6.5
30 d	67.0	3.4 (−1.3 to 8.1)	5.1	11.0 (4.1 to 17.9)	16.4	13.7 (5.1 to 22.3)	20.4
Acute care use in a year (rate per 100 patients with SMI)							
ED visits not leading to hospitalization	15.1	0.2 (−0.3 to 0.7)	1.4	1.0 (0.2 to 1.7)	6.4	0.5 (−0.5 to 1.5)	3.4
Hospitalization	6.0	0.2 (0.1 to 0.3)	2.9	0.4 (0.3 to 0.6)	7.1	0.5 (0.3 to 0.7)	7.6
Medication treatment							
No. of months in year of appropriate medication per person	5.9	−0.01 (−0.05 to 0.05)	−0.11	−0.02 (−0.08 to 0.04)	−0.39	−0.03 (−0.10 to 0.05)	−0.47

Abbreviations: ED, emergency department; SMI, serious mental illnesses.

^a^ Telemental health service visits per patient with serious mental illness in county in 2018.

### Subgroup Analyses

Our findings were qualitatively similar in our sensitivity analysis using a prospective cohort and when we examined the findings among individuals younger than 65 years vs those aged 65 years or older. In our subgroup analysis comparing those dual-eligible and non–dual eligible for Medicaid, we observed that the association of telehealth service use with visit patterns were similar. However, medication adherence for non–dual eligible patients was higher with high-telemedicine uptake vs no-telemedicine uptake by 0.49 months (95% CI, 0.31-0.67 months) (16% relative increase), but there was no significant difference among those with dual eligibility, with a differential of −0.01 months (95% CI, −0.02 to −0.09 months) (−0.21% relative increase) between the high- and low-uptake groups (Table 3).

**Table 3.** Subgroup and Sensitivity Analyses

Variable	County categories^a^						
	None (0)	Low (0-0.11)		Moderate (0.11-0.30)		High (>0.30)	
	Reference rate in 2010 in no use, %	% Difference vs reference group (95% CI)	% Change vs no use group	% Difference vs reference group (95% CI)	% Change vs no use group	% Difference vs reference group (95% CI)	% Change vs no use group
**Main regression**							
≥1 Visit per quarter in year, %	15.1	0.4 (0.1 to 0.6)	2.3	0.02 (−0.30 to 0.33)	0.1	1.2 (0.8 to 1.6)	8.0
≥1 Visit per semester in year, %	27.7	0.4 (0.2 to 0.6)	1.4	0.7 (0.4 to 1.1)	2.5	1.3 (0.9 to 1.8)	4.7
No. of months in year of appropriate medication per person	5.9	−0.01 (−0.05 to 0.05)	−0.11	−0.02 (−0.08 to 0.04)	−0.39	−0.03 (−0.10 to 0.05)	−0.47
**Non–dual eligibility**							
≥1 Visit per quarter in year, %	8.8	0.3 (0.3 to 0.4)	3.4	0.8 (0.7 to 0.9)	9.1	0.20 (−0.02 to 0.42)	2.3
≥1 Visit per semester in year, %	19.2	0.6 (0.5 to 0.7)	3.1	1.4 (1.3 to 1.6)	7.3	3.1 (2.7 to 3.4)	16.1
No. of months in year of appropriate medication per person	3.0	0.09 (0.05 to 0.13)	2.9	0.22 (0.13 to 0.31)	7.4	0.5 (0.3 to 0.7)	16.3
**Dual eligibility**							
≥1 Visit per quarter in year, %	21.8	0.3 (0.2 to 0.4)	13.8	0.1 (0.0 to 0.3)	0.5	1.2 (1.1 to 1.4)	5.5
≥1 Visit per semester in year, %	45.8	0.5 (0.4 to 0.6)	1.1	0.5 (0.3 to 0.7)	1.1	1.4 (1.2 to 1.6)	3.1
No. of months in year of appropriate medication per person	6.8	−0.04 (−0.01 to −0.02)	−0.62	−0.04 (−0.08 to −0.07)	−0.63	−0.01 (−0.02 to −0.09)	−0.21
**Age <65 y**							
≥1 Visit per quarter in year, %	16.0	0.3 (0.2 to 0.4)	2.8	0.1 (−0.1 to 0.2)	0.8	1.3 (1.1 to 1.5)	8.1
≥1 Visit per semester in year, %	28.1	0.3 (0.2 to 0.4)	1.1	0.5 (0.3 to 0.6)	1.6	1.5 (1.4 to 1.5)	5.3
No. of months in year of appropriate medication per person	6.0	−0.01 (−0.18 to 0.32)	−0.12	−0.01 (−0.03 to 0.50)	−0.22	0.5 (0.5 to 0.6)	8.3
**Age ≥65 y**							
≥1 Visit per quarter in year, %	9.1	0.3 (0.2 to 0.3)	9.1	0.6 (0.5 to 0.7)	6.3	1.3 (1.1 to 1.5)	14.3
≥1 Visit per semester in year, %	19.8	0.5 (0.5 to 0.6)	2.6	1.1 (1.0 to 1.3)	5.6	2.2 (1.9 to 2.4)	11.1
No. of months in year of appropriate medication per person	5.3	−0.1 (−0.2 to −0.6)	−1.9	0.05 (−0.05 0.14)	0.85	0.09 (−0.06 to 0.24)	1.7
**Prospective cohort**							
≥1 Visit per quarter in year, %	19.0	0.4 (0.2 to 0.6)	2.1	0.17 (−0.08 to 0.40)	0.89	0.9 (0.6 to 1.2)	4.8
≥1 Visit per semester in year, %	34.0	0.3 (0.1 to 0.5)	0.9	0.4 (0.1 to 0.7)	1.3	1.0 (0.7 to 1.4)	2.9
No. of months in year of appropriate medication per person	6.0	−0.01 (−0.02 to 0.02)	−0.01	−0.03 (−0.06 to 0.003)	−0.53	−0.04 (−0.08 to −0.00)	−0.67

^a^ Telemental health service visits per patient with serious mental illness in county in the year.

## Discussion

To our knowledge, this is the first systematic attempt to assess whether telemental health service uptake is associated with quality of care of patients with SMI outside clinical trials.^22,23,24^ Among patients with SMI in the Medicare program residing in nonmetropolitan counties from 2010 to 2018, we noted substantial increases in telemental health service use. Along with the large increase in telemental health service use in these counties, there was a substantial decrease in in-person care. In net, in counties with greater telemental health service uptake, we observed relative increases in the fraction of patients with SMI who received minimum levels of care. However, contrary to expectations, we generally observed an increase in hospitalizations and ED visits and no substantive improvements in medication adherence in these counties.

Our initial hypothesis assumed that use of telemental health visits would be additive to in-person care and therefore be a factor in more frequent contact with specialty mental health care professionals and, in turn, better medication adherence and fewer acute crises resulting in ED visits or hospitalizations. Our results do not support this hypothesis. We observed increases in patients receiving minimum levels of visits and follow-up care but did not observe better medication adherence or fewer acute crises.

The results instead support a different interpretation. Telemental health service largely substituted for in-person visits in the communities that embraced it. It is possible that nonmetropolitan counties that implemented telemental health service did so because of an increased unmet need for specialty mental health care professionals. For example, possibly a psychiatrist retired or left the community and telemental health service visits were used because recruitment of a local clinician to replace the one no longer there was difficult. If these counties had not turned to telemental health service, perhaps they would have experienced a substantial decrease in visits and decreases in quality of care.^11^ In this light, a positive interpretation of our findings is that shifting a substantial fraction of care to telemental health service appears to have no negative outcomes associated with the quality of care patients with SMI received. Earlier research has described the feasibility of applying telemedicine in the treatment of schizophrenia and psychotic disorders.^25^ Our findings extend the literature, supporting the idea that telemental health service can be a safe and similarly beneficial substitute for in-person care.

There is one cautionary note. Contrary to our initial hypothesis, we found that greater telemental health service use in a nonmetropolitan county was associated with small increases in hospitalizations and ED visits. This increase could be interpreted as a sign that telemental health service is associated with worse quality of care. Another explanation is that, to prevent hospitalizations and ED visits, one would need much greater use of outpatient care than we observed in this study along with substantial increases in medication adherence. Alternatively, it may be that our underlying assumption that, at a population level, more ED visits and hospitalizations are a sign of poor quality of care is incorrect. In some circumstances this greater need for ED visits and hospitalizations might be a sign of better care and that patients in acute crisis were correctly identified and stabilized.

### Limitations

This study has limitations. First, uptake of telemental service is not random. Although we studied the results of changes in quality outcomes associated with increased use of telemental health service within a county, it is possible that there are other changes over time in a given county that could have affected the changes we observed. Second, on a related note, we do not know what factors may have affected the increase in the use of telemedicine service. There could be other reasons that telemental health service use increased, such as increases in demand owing to stressors in a community or introduction of broadband internet in the county. Third, our claims data do not capture visits by licensed professional counselors or licensed marriage and family therapists among patients dually insured by Medicaid. These visits are not reimbursed by Medicare but are reimbursed by Medicaid in a few states. However, we did not find any differential associations between telemedicine use by visits among those with and without Medicaid. Inclusion of dually eligible Medicare enrollees is consistent with earlier literature.^4,5,26,27^ Fourth, among the subgroup of Medicare beneficiaries not dually eligible for Medicaid, we found an association between greater telemental health service use and greater medication adherence. What underlies that association in just this subgroup of patients is unclear and merits further investigation. Fifth, our sample was limited to the nonmetropolitan Medicare population of patients with SMI. Many of these patients are disabled and have severe disease; further studies are needed to expand data to broader SMI populations. Sixth, it is not clear at what level of substitution quality problems might be affected by greater use of telemental health service. In our research, roughly a quarter of specialty visits were provided via telemental health service in counties with high use of telemental health service, but there may be a different outcome if there was even greater use.

## Conclusions

In this cohort study, at a county level, among nonmetropolitan beneficiaries with SMI, we found that greater use of telemental health service was associated with modest increases in contact with outpatient specialty mental health professionals and a greater likelihood of follow-up after hospitalization. However, no substantive changes in medication adherence were noted and an increase in hospitalizations was observed.

## Notes

Supplement. **eTable 1.** List of Antipsychotic/Antimanic Drugs **eTable 2.** Supplemental Data Dictionary for Table 1 **eFigure.** Regression Model **eTable 3.** List of HCPCS Codes for Defining Outpatient Visits

## References

[1] Alegría M, Canino G, Ríos R, . Inequalities in use of specialty mental health services among Latinos, African Americans, and non-Latino Whites. Psychiatr Serv. 2002;53(12):1547-1555. doi:10.1176/appi.ps.53.12.1547 12461214

[2] Merwin E, Hinton I, Dembling B, Stern S. Shortages of rural mental health professionals. Arch Psychiatr Nurs. 2003;17(1):42-51. doi:10.1053/apnu.2003.1 12642887

[3] Lambert D, Hartley D. Linking primary care and rural psychiatry: where have we been and where are we going? Psychiatr Serv. 1998;49(7):965-967. doi:10.1176/ps.49.7.965 9661236

[4] Figueroa JF, Phelan J, Orav EJ, Patel V, Jha AK. Association of mental health disorders with health care spending in the Medicare population. JAMA Netw Open. 2020;3(3):e201210. doi:10.1001/jamanetworkopen.2020.1210 32191329 PMC7082719

[5] Germack HD, Bizhanova Z, Roberts ET. Substantial hospital level variation in all-cause readmission rates among medicare beneficiaries with serious mental illness. Healthc (Amst). 2020;8(3):100453. doi:10.1016/j.hjdsi.2020.100453 32919590 PMC7495504

[6] Mehrotra A, Chernew ME, Linetsky D, Hatch H, Cutler D, Schneider EC. The impact of COVID-19 on outpatient visits in 2020: visits remained stable, despite a late surge in cases. Commonwealth Fund. Published February 22, 2021. Accessed March 25, 2022. https://www.commonwealthfund.org/publications/2021/feb/impact-covid-19-outpatient-visits-2020-visits-stable-despite-late-surge

[7] García-Lizana F, Muñoz-Mayorga I. What about telepsychiatry? a systematic review. Prim Care Companion J Clin Psychiatry. 2010;12(2):PCC.09m00831. 20694116 10.4088/PCC.09m00831whi PMC2911004

[8] Chakrabarti S. Usefulness of telepsychiatry: a critical evaluation of videoconferencing-based approaches. World J Psychiatry. 2015;5(3):286-304. doi:10.5498/wjp.v5.i3.286 26425443 PMC4582305

[9] Hilty DM, Ferrer DC, Parish MB, Johnston B, Callahan EJ, Yellowlees PM. The effectiveness of telemental health: a 2013 review. Telemed J E Health. 2013;19(6):444-454. doi:10.1089/tmj.2013.0075 23697504 PMC3662387

[10] Center for Connected Health Policy. Understanding telehealth policy. Accessed March 25, 2022. https://www.cchpca.org/

[11] Patel SY, Huskamp HA, Busch AB, Mehrotra A. Telemental health and US rural-urban differences in specialty mental health use, 2010-2017. Am J Public Health. 2020;110(9):1308-1314. doi:10.2105/AJPH.2020.305657 32673109 PMC7427215

[12] US Department of Health and Human Services. Telehealth services: rural health fact sheet series. December 2014. Accessed May 25, 2022. https://www.kff.org/wp-content/uploads/sites/3/2015/06/telehealthsrvcsfctsht.pdf

[13] Mehrotra A, Huskamp HA, Souza J, . Rapid growth in mental health telemedicine use among rural Medicare beneficiaries, wide variation across states. Health Aff (Millwood). 2017;36(5):909-917. doi:10.1377/hlthaff.2016.1461 28461359

[14] Mehrotra A, Jena AB, Busch AB, Souza J, Uscher-Pines L, Landon BE. Utilization of telemedicine among rural Medicare beneficiaries. JAMA. 2016;315(18):2015-2016. doi:10.1001/jama.2016.2186 27163991 PMC4943212

[15] Lemke S, Boden MT, Kearney LK, . Measurement-based management of mental health quality and access in VHA: SAIL mental health domain. Psychol Serv. 2017;14(1):1-12. doi:10.1037/ser0000097 28134552

[16] Trafton JA, Greenberg G, Harris AH, . VHA mental health information system: applying health information technology to monitor and facilitate implementation of VHA Uniform Mental Health Services Handbook requirements. Med Care. 2013;51(3)(suppl 1):S29-S36. doi:10.1097/MLR.0b013e31827da836 23407008

[17] Fortney J, Sullivan G, Williams K, Jackson C, Morton SC, Koegel P. Measuring continuity of care for clients of public mental health systems. Health Serv Res. 2003;38(4):1157-1175. doi:10.1111/1475-6773.00168 12968822 PMC1360938

[18] National Quality Forum. 2021. Accessed March 25, 2022. https://www.qualityforum.org/Qps

[19] National Committee for Quality Assurance. HEDIS measures and technical resources. Accessed March 25, 2022. https://www.ncqa.org/hedis/measures

[20] Clancy CM. The persistent challenge of avoidable hospitalizations. Health Serv Res. 2005;40(4):953-956. doi:10.1111/j.1475-6773.2005.00442.x 16033486 PMC1361197

[21] Centers for Medicare & Medicaid Services. Medicare & Your Mental Health Benefits. 2022. Accessed March 25, 2022. https://www.medicare.gov/sites/default/files/2021-07/10184-Medicare-and-Your-Mental-Health-Benefits.pdf

[22] van den Berg N, Grabe HJ, Baumeister SE, Freyberger HJ, Hoffmann W. A telephone- and text message-based telemedicine concept for patients with mental health disorders: results of a randomized controlled trial. Psychother Psychosom. 2015;84(2):82-89. doi:10.1159/000369468 25721861

[23] Barnett P, Goulding L, Casetta C, . Implementation of telemental health services before COVID-19: rapid umbrella review of systematic reviews. J Med internet Res. 2021;23(7):e26492. doi:10.2196/26492 34061758 PMC8335619

[24] Talley RM, Brunette MF, Adler DA, . Telehealth and the community SMI population: reflections on the disrupter experience of COVID-19. J Nerv Ment Dis. 2021;209(1):49-53. doi:10.1097/NMD.0000000000001254 33003053

[25] Santesteban-Echarri O, Piskulic D, Nyman RK, Addington J. Telehealth interventions for schizophrenia-spectrum disorders and clinical high-risk for psychosis individuals: a scoping review. J Telemed Telecare. 2020;26(1-2):14-20. doi:10.1177/1357633X18794100 30134781

[26] McDowell A, Huskamp HA, Busch AB, Mehrotra A, Rose S. Patterns of mental health care before initiation of telemental health services. Med Care. 2021;59(7):572-578. doi:10.1097/MLR.0000000000001537 33797510 PMC8265030

[27] Roberto P, Brandt N, Onukwugha E, Stuart B. Redaction of substance abuse claims in Medicare research files affects spending outcomes for nearly one in five beneficiaries with serious mental illness. Health Serv Res. 2017;52(3):1239-1248. doi:10.1111/1475-6773.12528 27453380 PMC5441491

