# Supplementary material for: Association Between Telemedicine Use in Nonmetropolitan Counties and Quality of Care Received by Medicare Beneficiaries With Serious Mental Illness
Source: JAMA Netw Open. 2022 Jun 27;5(6):e2218730. doi: 10.1001/jamanetworkopen.2022.18730 (PMC9237790; doi:10.1001/jamanetworkopen.2022.18730)
Supplement: Supplement. — eTable 1. List of Antipsychotic/Antimanic Drugs eTable 2. Supplemental Data Dictionary for Table 1 eFigure. Regression Model eTable 3. List of HCPCS Codes for Defining Outpatient Visits [file jamanetwopen-e2218730-s001.pdf]

Supplementary Online Content

Wang B, Huskamp HA, Rose S, et al. Association between telemedicine use in nonmetropolitan counties and quality of care received by Medicare beneficiaries with serious mental illness. *JAMA Netw Open*. 2022;5(6):e2218730. doi:10.1001/jamanetworkopen.2022.18730

eTable 1. List of Antipsychotic/Antimanic Drugs

eTable 2. Supplemental Data Dictionary for Table 1

eFigure. Regression Model

eTable 3. List of HCPCS Codes for Defining Outpatient Visits

This supplementary material has been provided by the authors to give readers additional information about their work.

© 2022 Wang B et al. *JAMA Network Open.*

eTable 1. List of Antipsychotic/Antimanic Drugs

Generic Name	Type
ARIPIPRAZOLE	antipsychotic
ASENAPINE	antipsychotic
BREXPIPRAZOLE	antipsychotic
CARBAMAZEPINE	amanic
CARIPRAZINE	antipsychotic
CHLORPROMAZINE	antipsychotic
CHLORPROTHIXENE	antipsychotic
CLOZAPINE	antipsychotic
DIVALPROEX	amanic
FLUPHENAZINE	antipsychotic
HALOPERIDOL	antipsychotic
ILOPERIDONE	antipsychotic
LAMOTRIGINE	amanic
LITHIUM	amanic
LOXAPINE	antipsychotic
LURASIDONE	antipsychotic
MESORIDAZINE	antipsychotic
MOLINDONE	antipsychotic
OLANZAPINE	antipsychotic
OXCARBAZEPINE	amanic
PALIPERIDONE	antipsychotic
PERPHENAZINE	antipsychotic
PIMOZIDE	antipsychotic
PIPERACETAZINE	antipsychotic
PROMAZINE	antipsychotic
QUETIAPINE	antipsychotic
RISPERIDONE	antipsychotic
THIORIDAZINE	antipsychotic
THIOTHIXENE	antipsychotic
TRIFLUOPERAZINE	antipsychotic
TRIFLUPROMAZINE	antipsychotic
VALPROIC	amanic
ZIPRASIDONE	antipsychotic

© 2022 Wang B et al. *JAMA Network Open.*

eTable 2. Supplemental Data Dictionary for Table 1

Variable	Source	Year	Other Names	Definition
Household Income (Median $)	AHRF	2019	F1322608	County median household income.
Population Density (Person/Square Mile)	AHRF	2019	F1387600	Number of people per square mile in county.
Household with Broadband (%)	Census	2010		Percentage of households with broadband access in county.
Hospital Beds per 1,000 people in county	AHRF	2019	F0892107	Number of hospital beds per capita in county.
Community Health Centers per 1,000 people in county	AHRF	2019	F1322108	Number of community centers per capita in county.
Physicians/Advanced Practice Nurses per 100 people in county	AHRF	2019	F1322108, F1212908	Sum of total active MDs and total active APRNs in county.

© 2022 Wang B et al. *JAMA Network Open.*

eFigure. Regression Model

$$Y_i = County + Year + High_i{'}b + Moderate_i{'}r + Low_i{'}s$$

© 2022 Wang B et al. *JAMA Network Open.*

eTable 3. List of HCPCS codes for defining outpatient visits

H0001	H0002	H0004	H0005	H0006	H0014	H0015	H0016	H0020	H0022	H0023	H0028	H0030	H0031
H0033	H0034	H0036	H0037	H0038	H0039	H0040	H0046	H0049	H0050	H1011	H2000	H2001	H2010
H2013	H2014	H2015	H2016	H2017	H2018	H2019	H2020	H2021	H2023	H2024	H2025	H2026	H2027
H2028	H2020	H2030	H2031	H2032	H2033	H2037	H5010	H5020	H5025	H5030	H5220	H5230	H5240
H5299	M006<br>4	Q3014	S0280	S0281	S3005	S9110	S9127	S9454	S9482	T0006	T0011	T0012	T0015
T0016	T1017	T1018	T1023	T1024	T1025	T1026	T1027	T1040	T1041	T2010	T2011	T2012	T2013
T2014	T2015	T2018	T2019	T2020	T2021	T2022	T2023	T2036	T2037	Z0001	Z0002	G0071	G0072
G0073	G0074	G0075	G0076	G0077	G0078	G0079	G0080	G0081	G0082	G0175	G0351	G0396	G0397
G0436	G0437	G0442	G0443	G0463	G0466	G0467	G0469	G0470	G0502	G0503	G0504	G0505	G0506
G0507	G0511	G0512	G0513	G0514	G0515	0359T	0360T	0361T	0362T	0363T	0364T	0365T	0366T
0367T	0368T	0369T	0370T	0371T	0372T	0373T	0374T	90791	90792	90801	90802	90804	90805
90806	90807	90808	90809	90810	90811	90812	90813	90814	90815	90820	90831	90832	90833
90834	90835	90836	90837	90838	90839	90840	90842	90843	90844	90845	90846	90847	90848
90849	90853	90855	90857	90862	90865	90875	90876	90880	90900	90901	90902	90904	90906
90908	90910	97003	97004	99058	99201	99202	99203	99204	99205	99211	99212	99213	99214
99215	99241	99242	99243	99244	99245	99371	99372	99373	99404	99408	99409	99412	99420
99487	99489	99490	98966	98967	98968	G0438	G0439	G0468	G2025				

© 2022 Wang B et al. *JAMA Network Open.*
